# Extinction, coexistence, and localized patterns of a bacterial population with contact-dependent inhibition

**DOI:** 10.1186/1752-0509-8-23

**Published:** 2014-02-27

**Authors:** Andrew E Blanchard, Venhar Celik, Ting Lu

**Affiliations:** 1 Department of Physics, University of Illinois at Urbana-Champaign, 1110 West Green Street, 61801 Urbana, USA; 2 Department of Bioengineering, University of Illinois at Urbana-Champaign, 1304 West Springfield Avenue, Urbana IL 61801, USA; 3 Institute for Genomic Biology, University of Illinois at Urbana-Champaign, 1206 West Gregory Drive, Urbana IL 61801, USA

**Keywords:** Contact dependent inhibition, Bacterial population, Competition, Extinction and coexistence, Spatial aggregation

## Abstract

**Background:**

Contact-dependent inhibition (CDI) has been recently revealed as an intriguing but ubiquitous mechanism for bacterial competition in which a species injects toxins into its competitors through direct physical contact for growth suppression. Although the molecular and genetic aspects of CDI systems are being increasingly explored, a quantitative and systematic picture of how CDI systems benefit population competition and hence alter corresponding competition outcomes is not well elucidated.

**Results:**

By constructing a mathematical model for a population consisting of CDI+ and CDI- species, we have systematically investigated the dynamics and possible outcomes of population competition. In the well-mixed case, we found that the two species are mutually exclusive: Competition always results in extinction for one of the two species, with the winner determined by the tradeoff between the competitive benefit of the CDI+ species and its growth disadvantage from increased metabolic burden. Initial conditions in certain circumstances can also alter the outcome of competition. In the spatial case, in addition to exclusive extinction, coexistence and localized patterns may emerge from population competition. For spatial coexistence, population diffusion is also important in influencing the outcome. Using a set of illustrative examples, we further showed that our results hold true when the competition of the population is extended from one to two dimensional space.

**Conclusions:**

We have revealed that the competition of a population with CDI can produce diverse patterns, including extinction, coexistence, and localized aggregation. The emergence, relative abundance, and characteristic features of these patterns are collectively determined by the competitive benefit of CDI and its growth disadvantage for a given rate of population diffusion. Thus, this study provides a systematic and statistical view of CDI-based bacterial population competition, expanding the spectrum of our knowledge about CDI systems and possibly facilitating new experimental tests for a deeper understanding of bacterial interactions.

## Background

Bacteria are highly social and present dominantly in the form of complex communities where they interact through a variety of fashions [1-3]. Among all types of bacterial interactions discovered, competition has been identified as the most prevalent by recent studies [4-6]. The ubiquity of competition has been mainly attributed to the limited space and resources of natural environments. To maximize their survival and reproduction, bacteria have indeed developed numerous competition strategies, including interference competition where a species directly harms another via active production of toxin and other effectors [4,6-8].

Interference competition was initially shown to be mediated by diffusible soluble factors, such as antibiotics and bacteriocins. These effector molecules serve to potently decrease survival and reproduction of neighboring bacteria at a long range spatial scale [2,9-11]. For instance, Lactococcus lactis produces and secretes nisin, a small antimicrobial peptide, into the extracellular milium (e.g., milk) to efficiently inhibit other bacterial species for lactose competition [12]. A set of recent studies, however, have illustrated that interference competition can also occur through direct physical contact between cells, revealing a new class of competition with an interaction scale restricted to nearest neighbors [13-17].

Furthermore, studies have uncovered a surprisingly high degree of diversity among these contact–dependent inhibitions (CDIs). They occur across a wide range of organisms including both Gram- negative and positive bacteria [17-21], use different toxins similar to nuclease, tRNAse and DNase, and further exploit various delivery machineries spanning Type III, IV, V, and VI secretion systems [13-15,22,23]. Moreover, it has been shown that certain strains even have multiple CDI modules and multiple toxins for competition [17,24].

Despite their structural and compositional diversity, all of the CDI systems possess a common mode of action in which growth inhibition toxins are deployed into competitor cells via direct cell to cell physical contact. A representative example is the CdiBAI system discovered in the enterobacterium *E. coli* EC93 [14]: The system consists of three functional components: CdiA, the toxin effector, CdiB, the *β*-barrel protein localized to the outer membrane for effector export, and CdiI, the immunity protein. Upon contact with a neighboring cell, a CDI equipped cell employs CdiB to inject toxin CdiA into the target cell to inhibit its growth while expressing the immunity protein CdiI to prevent autoinhibition.

The intriguing functionality and characteristics of CDI motivate us to ask the following questions: How does CDI impact the competition between a CDI equipped (CDI+) and deficient (CDI-) species? Can the two species coexist, or does extinction always occur for one of the species? Due to the intrinsic association of protein expression and metabolic load, will the growth disadvantage of CDI+ species counteract its competition advantage from toxin production? How does cell motility alter the competition outcome in different environmental settings, such as liquid or solid agar?

Although current experimental efforts [13,23-26] have started to address some of the above questions, a systematic and quantitative understanding of CDI-based bacterial competition has not been achieved. In particular, it is not clear how the competition outcome is influenced by the inhibition advantage of CDI-based competition and the growth disadvantage associated with metabolic load. Moreover, most experimental efforts have primarily focused on liquid culture settings where populations are well mixed [14,24,25] but little has been elucidated when competitions occur in space. There is hence a clear need for a systematic investigation of CDI-based bacterial competition that integrates the tradeoff between competitor inhibition and metabolic cost with spatiotemporal dynamics.

Here, we present a mathematical model to describe a bacterial population with CDI+ and CDI- species that compete through both contact-dependent inhibition and nutrient utilization. With this model, we studied the composition of the competing population in the well-mixed case, showing that the outcome is always extinction for one of the species depending on initial conditions as well as the tradeoff between inhibition strength and metabolic cost. We then continued to investigate the dynamics of the population in one dimensional space, revealing possible spatial coexistence of the species through aggregation. To acquire a statistical understanding of the coexistence patterns, we further conducted a systematic survey into the spatial structure of the competing populations by altering the interplay between inhibition strength and metabolic growth disadvantage. A set of illustrating tests in two dimensional space was also implemented to demonstrate the generality of our results. We finally conclude by summarizing our findings and discussing possible future developments.

## Results

### A mathematical model of bacterial competition with CDI

Bacterial competition has been modeled through coupled systems of ordinary differential equations, dating back to the work of Lotka and Volterra [27,28]. Later developments of the original Lotka-Volterra model have incorporated the effects of nutrient limitation and species diffusion [29-36]. Typically, these models incorporated only on-site interactions (i.e. the interaction range for competing species is taken to be infinitesimally small). For bacteria competing through CDI, however, it is natural to explicitly include a finite interaction range in order to account for the intrinsic nearest-neighbor effects of toxin injection in the system.

Previous studies on competition with a finite interaction range have shown that spatial aggregation is possible in the long time limit [37-43]. For instance, a single species with nonlocal interactions has unstable homogeneous states for certain functional forms of its growth rate, leading to a spatial distribution of the population with clumps of high species concentration separated by regions with low density [41,44-47]. For two species systems, it has been shown that systems with specific features, such as systems with Allee effects and completely symmetric interactions, may have steady spatial structures [37,40,48]. Despite these advances, a mathematical model appropriate for bacterial populations with the nearest neighbor and asymmetric interactions implemented through CDI has not been investigated.

To enable a quantitative and systematic investigation of CDI-based bacterial competition, we constructed a mathematical model that describes a two-species population with one CDI+ (CDI equipped) and the other CDI- (CDI deficient) as follows:

(1) $$\begin{array}{lll} \;\frac{\text{du}}{\text{dt}} & =u\left(\alpha -u-v-c\int f(|\chi -x|)v(\chi )\mathrm{d\chi}\right)+D_{u}\nabla^{2}u\; & \;\\ \;\frac{\text{dv}}{\text{dt}} & =v(\beta -u-v)+D_{v}\nabla^{2}v\; \end{array}$$

where *u* and *v* are the concentrations of the CDI- and CDI+ species respectively. This model incorporates the asymmetrical interaction from the CDI+ to CDI- species through the integral term (-uc∫f(|χ-x|)v(χ)dχ), the effects of limited shared resources through the logistic growth terms, and cell motilities through the corresponding diffusion terms. Here, nondimensionalization has been implemented with the original model detailed in Additional file 1: Section 1.

Due to the intrinsic direct physical contact of CDI, bacterial interactions follow a discrete, nearest neighbor fashion, i.e., *f*(|*x*_*i*_ - *x*_*j*_|) = 1 / 3*δ* when *j* = *i* + 1, *i*, *i* - 1 and *f*(|*x*_*i*_ - *x*_*j*_|) = 0 otherwise. The above model can thus be discretized and rewritten in one spatial dimension as:

(2) $$\begin{array}{lll} \frac{{\text{du}}_{i}}{\text{dt}} & =u_{i}(\alpha -u_{i}-v_{i}-c_{1}(v_{i+1}+v_{i}+v_{i-1}))\; & \;\\ & \;+\frac{D}{\delta^{2}}(u_{i+1}-2u_{i}+u_{i-1})\; & \;\\ \frac{{\text{dv}}_{i}}{\text{dt}} & =v_{i}(\beta -u_{i}-v_{i})+\frac{D}{\delta^{2}}(v_{i+1}-2v_{i}+v_{i-1})\;\\ \;i & =1,2,\ldots ,N\; & \; \end{array}$$

where *δ* is the grid spacing, *u*_*i*_ (*v*_*i*_) is the concentration of the CDI- (CDI+) species in space *iδ*, N is the number of total grid points, and *D* is the diffusion constant (*D*_*u*_ = *D*_*v*_ = *D* assumed for simplicity). In addition, we have used *c*_1_ = *c* / 3 to reflect the fact that there are three interaction locations for CDI in one dimension including one on–site and two nearest neighbors. Furthermore, *c*_1_ is a constant here, corresponding to a constitutive expression of the CDI system as seen in certain species such as *E. coli* EC93 [24]. Periodic boundary conditions are imposed so that *u*_*N*+1_=*u*_1_ and *u*_0_ = *u*_*N*_ (similarly for *v*).

Although this model is generic and does not describe biochemical details of the competing population, it captures the main biological features of the system in realistic settings: (1) Cellular growth is described using a logistic growth model, which accounts for nutrient limitation in experimental settings and has been adopted widely by previous theoretical studies [35]; (2) Cellular movement is modeled with a random diffusion term, which is adequate to describe non-motile cells with passive diffusion or motile cells without chemotaxis; (3) Constant growth inhibition of the CDI system is modeled, which is supported by experimental evidence showing that certain bacterial species have constitutive production of the CDI machinery [24].

Our model has four key parameters, namely *α*, *β*, *c*_1_, and *D*, which describe the growth rates of the CDI- and + species, inhibition strength from the CDI+ to CDI- species, and the motility of both species respectively. It is evident that if the CDI+ species (*v*) has a larger growth rate than the CDI- species (*u*) (i.e., *α*/ *β*<1), the outcome of competition will be the extinction of the CDI- species due to the additive combined affects of slower growth and nearest neighbor inhibition. However, the outcome is unclear when the metabolic load of producing proteins involved in CDI causes *α*/ *β*>1. We are thus motivated to understand how the tradeoff between inhibition strength and growth disadvantage influences the outcome of competition and what kinds of possible competition outcomes may result. In the following, we consider the tradeoffs between relative growth advantage and inhibition over a range of cell motility.

### Well-mixed case

We begin our investigation by considering the well–mixed case for our system, corresponding to a liquid assay or *D* → *∞* in the model. In this case, the solutions are assumed to be homogenous in space so that *u*_*i*_ = *u* and *v*_*i*_ = *v* for all *i*. By eliminating the spatial interactions of the system, we get an inhibition contribution of 3*c*_1_*v* (*cv*) to the growth rate of *u* at each site. The resulting equations are:

(3) $$\begin{array}{lll} \frac{\text{du}}{\text{dt}} & =u(\alpha -u-v(1+c))\; & \;\\ \frac{\text{dv}}{\text{dt}} & =v(\beta -u-v)\; \end{array}$$

from which we find four steady states, including (*u*, *v*) = (0, 0), (*u*, *v*) = (*α*, 0), (*u*, *v*) = (0, *β*), and (*u*, *v*) = (*β* - (*α* - *β*) / *c*, (*α* - *β*) / *c*).

**Figure 1:**
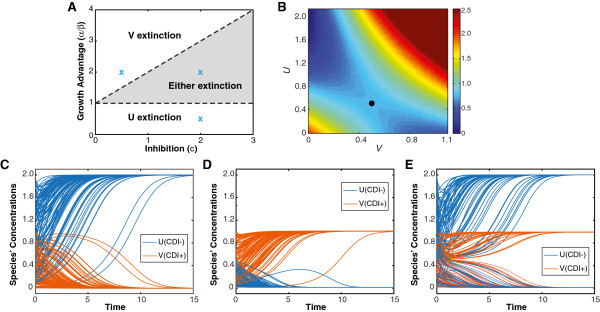
**Well-mixed competition of a bacterial population consisting of a CDI+ and a CDI- species. (A)** Phase diagram of the competing population. Grey region: competition of the population may lead to the extinction of one of the two species, depending on the initial conditions of the system. Upper white region: the CDI+ species (*v*) always goes extinct regardless of initial conditions. Lower white region: the CDI- species (*u*) always goes extinct regardless of initial conditions. **(B)** Plot of the Lyapunov function [36] of the competing population in the regime when extinction is possible for either species. The parameter set here corresponds to the blue cross in the grey region of Panel A. Notice that the minima for the system lie on the respective axes, showing two possible extinctions. The dark circle is the saddle point of the system. **C-E.** Sample time course trajectories of the two competing species. **(C)** Species *u* (CDI-) always dominates when growth advantage outweighs inhibition. Here, *α* / *β* = 2 and *c* = 0.5, corresponding to the cross in the upper white region of Panel A. **(D)** Species *v* (CDI+) always take over the population when it has a growth advantage and exerts inhibition. Here, *α* / *β* = 0.5 and *c* = 2.0, corresponding to the cross in the lower white region of Panel A. **(E)** Both of the species may dominate depending on their initial conditions. Here, *c* = 2.0 and *α* / *β* = 2.0, corresponding to the cross in the grey region of Panel A. In each panel (C-E), 100 pairs of trajectories (orange: CDI+, blue: CDI-) with random initial conditions are plotted.

The linear stability of these homogeneous steady states changes as we vary the parameters of the model due to the tradeoff between inhibition strength *c* and relative growth advantage *α* / *β* (Figure 1A). When the growth advantage of species *u* (CDI-), given as *α* / *β*, greatly outweighs the inhibition constant *c* (the upper white region of Figure 1A), species *u* drives *v* to extinction regardless of the initial conditions as illustrated in Figure 1C. Conversely, when the growth advantage of species *u* is less than one (the lower white region of Figure 1A), species *v* always drives species *u* to extinction (Figure 1D). There does exist, however, a certain parameter regime, namely 1 < *α* / *β* < 1 + *c* (the shaded region of Figure 1A), in which the outcome of the competition depends on the initial conditions (Figure 1E). In this parameter regime, both of the extinction states are linearly stable. A plot of the Lyapunov function [36] (Figure 1B) indeed shows two minima for the phase space of the system, corresponding to possible stable extinction states.

The above results suggest that the tradeoff between inhibition strength, due to the production of toxins by CDI, and relative growth advantage, due to the metabolic load associated with CDI, determines the possible outcomes of a two-species competition. This conclusion is in agreement with a previous experimental study concerning bacteriocin production [49], where initial concentrations of a competing population determined the extinct species. Although a diffusible toxin instead of CDI was employed in the experiment, it clearly supports our modeling results. In another experimental report, it was shown that a CDI+ strain is indeed capable of driving a CDI- strain toward extinction in a well-mixed environment [14]. In the future, it will be valuable to design experiments to systematically investigate the impacts of initial concentrations and metabolic load on the outcome of CDI-based species competition.

The dependence of competition outcomes on initial conditions in certain parameter regions may have additional important implications: It indicates that spatial aggregation and localized patterns may occur in space with appropriate initial conditions when the diffusion of the system is small.

### Phase diagram for the existence of spatial patterns

**Figure 2:**
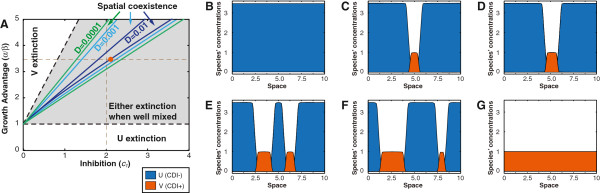
**Representative time course evolutions of the competing two-species population in one dimensional space with a growth advantage (*α*/*β*) of 3.5 and an inhibition (*c***_**1**_**) of 2.1.** Coexistence of the CDI+ and CDI- species may appear in certain parameter sets (Panel **A** and **B**). However, localized patterns will merge and eventually disappear with the increase of the diffusion constant for both species (Panel **A**: *D* = 0.001, Panel **B**: *D* = 0.01, and Panel **C**: *D* = 0.1). The same set of initial conditions are used for all of the panels. Here, only the spatiotemporal distributions of the concentration of species *u* (CDI-) are shown in the panels due to the mutual exclusion features of the two competing species. Those of the other species (CDI+, *v*) are available in Additional file 1: Figure S1–3.

In a preliminary search for the existence of stable patterns in one spatial dimension, we performed simulations with random initial conditions for a specific set of the growth advantage (*α* / *β*), inhibition (*c*_1_ = *c* / 3), and diffusion constants (*D*_*u*_ = *D*_*v*_ = *D*). Figure 2 illustrates three time courses in which the concentration distribution of species *u* shows stable localized patterns (spatial distribution of species *v* is anti-correlated with that of *u* and presented in Additional file 1: Figure S1–3). This phenomenon originates from the stability of both extinction states of our system in a certain parameter regime. Figure 2 further shows that, as the diffusion constant changes, the basin of attraction for the states may vary: as the diffusion constant is changed from 10^-3^ to 10^-2^ and to 10^-1^, the small stripes in Figure 2A become unstable in Figure 2B and all stripes become unstable in Figure 2C. Therefore, diffusion tends to destroy possible species aggregations within the population.

To systematically explore possible outcomes of the competition in space and to understand the conditions under which corresponding patterns emerge, we computationally examined possible steady states of the model as we varied the relative growth advantage (*α*/ *β*), inhibition strength (*c*_1_), and species motility (*D*) for different initial conditions. In principle, a parameter search can be implemented by repeatedly simulating our mathematical model (Eq. 2) with massive random initial conditions for each possible parameter combination and then by analyzing the corresponding steady states of the system. To reduce the computational cost of the problem, we chose a representative set of initial conditions for simulation in practice. Here, the initial condition sets consisted of two non-overlapping domains with each solely occupied by one of the two species at the system’s carrying capacity, i.e., grids 1-5 are solely occupied by *u* while the remaining space, grids 6-32, are fully occupied by *v* for the 32-grid space (detailed in Methods and Additional file 1: Figure S8).

**Figure 3:**
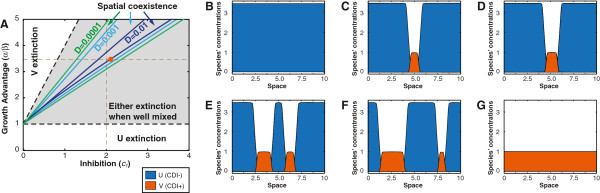
**Phase diagram and representative patterns of the competing population. (A)** Phase diagram. The grey shaded region corresponds to the case when both CDI+ or - extinctions are possible when the population is well mixed, identical to the grey region in Figure 1A. The green, blue and navy lines are the phase boundaries between spatial coexistence (beak regions) and extinction (outside of beak regions) when the rate of population diffusion is 0.0001, 0.001, and 0.01 respectively. **(B-G)**The most likely localized patterns of the competing population with different growth advantages and inhibitions. 10^4^ random initial conditions are used here as we move across the phase diagram with a growth advantage of 3.5, a diffusion constant of 10^-3^, and an inhibition varying from 1.7 to 2.3. **(B)** For an inhibition of 1.7, patterns may be present depending on initial conditions, but the most likely state is the extinction of species *v*. **(C-D)** For an inhibition of 1.8 and 1.9, the most likely pattern changes to a single stripe configuration. **(E-F)** For an inhibition of 2.0 and 2.1, the most likely pattern changes to a double stripe configuration. **(G)** For an inhibition of 2.3, only the extinction states are observed, with the most likely state being the extinction of *u*.

Figure 3A shows the phase diagram for the competition outcome of the population obtained from our numerical study. As illustrated in the figure, the beak regions outlined by the color lines are the coexistence parameter space where spatial localized patterns may emerge and be stable. The colors of the lines (green, cyan, and navy) correspond to the species’ diffusion constant of 0.0001, 0.001, and 0.01 respectively. Outside the beak regions, the competition of the population always lead to the extinction of one of the two species. Although obtained using the representative sets of initial conditions, the phase boundaries were tested and confirmed by employing 10^4^ random initial conditions for *D* = 0.001 with *α* / *β* ∈ [3,4]. To further illustrate the diversity of possible competition outcomes, representative patterns with the highest likelihood from random initial conditions are shown in Figure 3B–G, where the inhibition strength is varied across the phase diagram (*c*_1_ = 1.7, 1.8, 1.9, 2.0, 2.1, 2.3) for a fixed growth advantage (*α* / *β* = 3.5) and diffusion constant (*D* = 0.001).

It is important to notice that the spatial coexistence region is within the parameter region where both extinction states remain stable (shaded region), indicating that the emergence of patterns is not due to a diffusion driven instability of the homogeneous states like Turing patterns [35,50]. In addition, as every spatial aggregate of the two species within a stable pattern is locally similar to the extinction states of the system in the well-mixed case, the stability of both extinction states appears to be a necessary condition for the existence of spatial patterns. Thus, the tradeoff between relative growth advantage and inhibition strength continues to play a key role in determining possible outcomes of competition in space.

### Statistics of localized patterns

**Figure 4:**
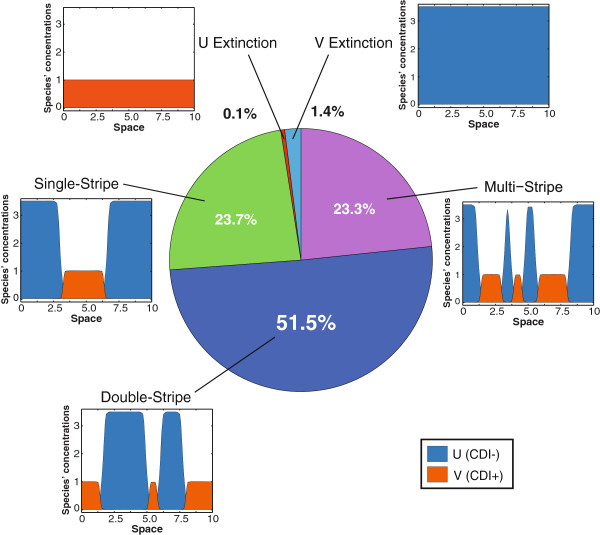
**Occurrence of localized patterns of the competing population with a growth advantage of 3*.*5, an inhibition of 2*.*2 and a diffusion constant of 10**^**-3**^**.** Various patterns may emerge in this parameter set, among which double-stripe patterns are the most dominant (51.5*%*), followed by single-stripe (23.7*%*) and then multi-stripe (23.3*%*) patterns. Only about 1.5*%* of initial conditions result in extinction for one of the species. Here, 10^4^ runs with random initial conditions are used to generate the statistics.

Through our computational survey of competition outcomes, we have found that diverse patterns may emerge from the competing population for a single parameter set: As illustrated in Figure 4, the competition of the population can possibly produce single-stripe, double-stripe, and multi-stripe localized patterns as well as the extinction of either species for a growth advantage of 3.5, inhibition constant of 2.2, and a diffusion constant of 10^-3^. Although various patterns were observed, their prevalence was different: In this specific case, double, single and multi–stripe patterns constitute the vast majority of possible outcomes with a relative abundance of 51.5*%*, 23.7*%*, and 23.3*%* respectively.

**Figure 5:**
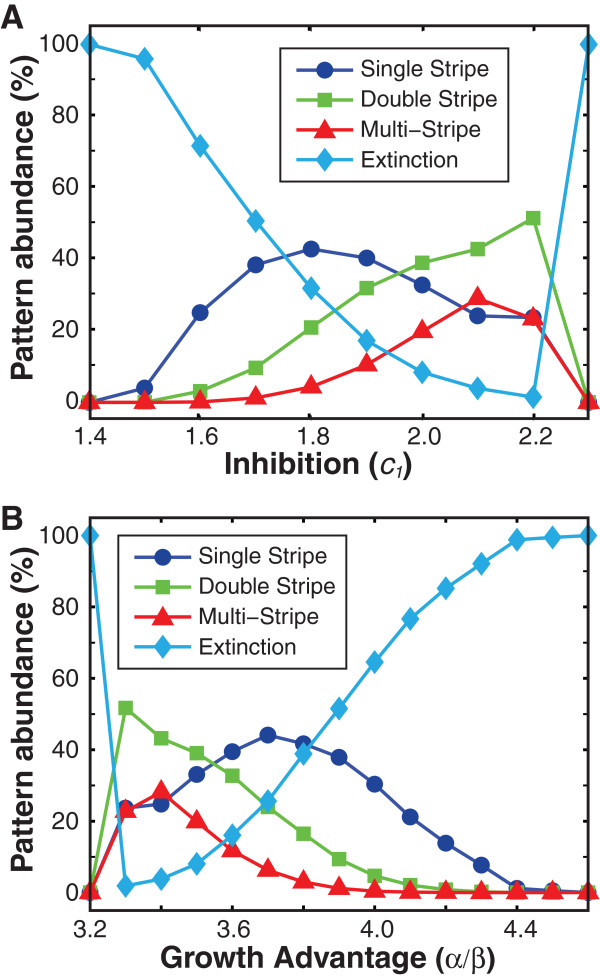
**Statistics of localized patterns as a function of growth advantage and inhibition.(A)** Relative abundance of spatial patterns as a function of CDI inhibition (*c*_1_) for a given growth advantage (*α*/*β* = 3.5). The most likely steady pattern transits from CDI+ extinction to single to double stripe, with a increasingly high prevalence of multi-stripes, as CDI inhibition increases. It makes a sharp change back to extinction (CDI-) as the parameters exit the spatial coexistence region. **(B)** Relative abundance of spatial patterns as a function of growth advantage (*α*/*β*) for a given CDI inhibition (*c*_1_ = 2.0). An opposite trend of pattern statistics is observed compared with panel A.

To reveal how key system parameters influence the occurrences of possible patterns, we performed the simulations of our model with 10^4^ random initial conditions for a varying inhibition strength (relative growth advantage and diffusion constant are fixed) and analyzed the corresponding statistics. As shown in Figure 5A, competition of the population always gives rise to extinction (species *v*) when the inhibition strength is weak (*c*_1_ = 1.4), corresponding to the left of the coexistence region in Figure 3A. However, as the inhibition strength increases, the system enters the coexistence parameter space and various patterns are observed with different relative occurrences. The competition outcome of the population becomes exclusively extinction (mostly of species *u*) when the inhibition is beyond a threshold (the right boundary of the coexistence region). Accordingly, the most probable pattern is changed from extinction to single-stripe patterns and later to double-stripe patterns before eventually returning back to extinction (Figure 3B-G). The observed alteration of pattern diversity and relative abundance is due to the fact that the orchestration of the tradeoff between inhibition and growth advantage is mandatory for the spatial coexistence of the two species and imbalance of the tradeoff results in the increase of species extinction. In addition to the relative abundance, the widths of stripes of localized patterns also change with the inhibition strength as shown in Table 1 (and Additional file 1: Table S3). In contrast to the relative abundance of pattern occurrences, pattern widths are less subjective to parameter changes, mainly due to the requirement of minimal size of localized patterns for stabilization and the mutual exclusion feature of the two species.

**Table 1:** **Summary of statistics for different inhibition values (*c***_**1**_**) using a growth advantage (*α*/*β*) of 3** ***.*** **5 and a diffusion constant (*D*) of 0** ***.*** **001**

***c***_**1**_	**Single stripe width**	**Double stripe width**	**Multi–stripe width**
1.4000	N/A	N/A	N/A
1.5000	8.9207 ±0.2727	3.9583 ±1.2969	0.0000
1.6000	9.1718 ±0.3142	4.1944 ±2.1219	2.5625 ±1.3797
1.7000	9.0973 ±0.4039	4.1153 ±2.0673	2.4557 ±1.4078
1.8000	8.9883 ±0.5083	4.0300 ±2.1113	2.3128 ±1.3519
1.9000	8.8421 ±0.6373	3.9168 ±2.0163	2.2297 ±1.2819
2.0000	8.5869 ±0.8970	3.7419 ±1.9762	2.0780 ±1.2299
2.1000	8.1909 ±1.2791	3.4835 ±1.8674	1.9343 ±1.1349
2.2000	6.7693 ±2.1881	3.0211 ±1.5841	1.8590 ±0.9125
2.3000	N/A	N/A	N/A

Widths are the average size of domains for species *u* in a system of length (*L* = 10) with the standard deviations shown.

Similar to the inhibition strength, the relative growth advantage can also considerably alter the localized patterns of the competing population. As illustrated in Figure 5B, the relative abundances of stable patterns vary with the growth advantage with the most probable pattern changing from extinction to double-stripe to single-stripe and finally to extinction again. This trend of pattern alterations is opposite to the case of varying inhibition strength (Figure 5A). This is primarily due to the opposite impacts of growth advantage and inhibition in shaping population structures. The statistics of the width of stripes of stable patterns with different growth advantages are shown in Table 2 (and Additional file 1: Table S4).

**Table 2:** **Summary of statistics for different growth advantage values (*α*/*β*) using an inhibition (*c***_**1**_**) of 2** ***.*** **0 and a diffusion constant (*D*) of 0** ***.*** **001**

***α*/*β***	**Single stripe width**	**Double stripe width**	**Multi–stripe width**
3.2000	N/A	N/A	N/A
3.3000	6.6860 ±2.2685	3.0043 ±1.5822	1.8632 ±0.9081
3.4000	8.2396 ±1.2199	3.4892 ±1.8759	1.9354 ±1.1538
3.5000	8.6220 ±0.8392	3.7115 ±1.9583	2.0711 ±1.2085
3.6000	8.8299 ±0.6749	3.8809 ±2.0082	2.1776 ±1.2621
3.7000	8.9415 ±0.5602	3.9947 ±2.0408	2.2990 ±1.3339
3.8000	9.0544 ±0.4490	4.0685 ±2.0718	2.3592 ±1.3373
3.9000	9.1103 ±0.3828	4.1275 ±2.0864	2.4225 ±1.4374
4.0000	9.1619 ±0.3433	4.1917 ±2.0968	2.5129 ±1.3891
4.1000	9.1953 ±0.2962	4.1774 ±2.1287	2.5104 ±1.4332
4.2000	9.2119 ±0.2598	4.2721 ±2.1072	2.5651 ±1.2789
4.3000	9.2310 ±0.2421	4.1741 ±2.3164	0.0000
4.4000	8.9557 ±0.2050	0.0000	0.0000
4.5000	8.9623 ±0.2083	0.0000	0.0000
4.6000	N/A	N/A	N/A

Widths are the average size of domains for species *u* in a system of length (*L* =10) with the standard deviations shown.

The statistics of the stripe patterns further show that the CDI- strain tends to have larger cluster sizes than the CDI+ strain on average within the coexistence region. In fact, for all coexistence statistics computed, the CDI- strain occupies a greater percentage of the space than the CDI+ strain. The disparity of the stripe widths for CDI+ and CDI- is primarily due to the non-local nearest neighbor interactions: To have a sustained domain, CDI- cells must build a buffer region to protect themselves when they are surrounded by CDI+ cells; in contrast, when CDI+ cells are surrounded by CDI- cells, no buffer layers are needed. Therefore, there is an increase in colony width for CDI- colonies, compared to CDI+ colonies. Importantly, the stripe width disparity does not exist in systems with only on–site interactions as employed previously in many competition studies [29-34]. Instead, both species will occupy the space equally on average. From an ecological perspective, the disparity of localized patterns shows the importance of non-local interactions in determining the species aggregation of bacterial communities and may offer new insights into the diversity of microbes.

### Perturbative expansion for patterned states

To gain insight into the exact requirements for inhibition and growth advantage necessary to produce spatial coexistence of the population, we have employed a perturbation approach in addition to the numerical results presented above. The perturbation approach is built around finding a stable spatially inhomogeneous steady state for the model with *D* = 0 and then considering how the state is perturbed when slow diffusion is allowed (see Additional file 1: Section 2 for details).

When *D* = 0, each equation in our model (Eq. 2) at steady state becomes the product of two linear equations. Thus, there are 2^2*N*^ possible steady states in total, where *N* is the number of grid points. To determine the possibility of coexistence, we consider the simplest possible steady state with coexistence and aggregation, in which species *u* and *v* each form a single domain at the respective carrying capacities with two single-grid-point transition layers connecting the two domains in space. By plugging this trial state into our model, we have:

(4) $$\begin{array}{lll} u_{i}=\alpha & v_{i}=0 & i=1,\ldots ,\frac{N}{2}-1\\ u_{i}=\alpha -\beta c_{1} & v_{i}=0 & i=\frac{N}{2}\\ u_{i}=0 & v_{i}=\beta & i=\frac{N}{2}+1,\ldots N-1\\ u_{i}=\alpha -\beta c_{1} & v_{i}=0 & i=N \end{array}$$

for which we have assumed that two transition layers occur at *i* = *N* / 2 and *i* = *N* for simplicity (our analysis can be similarly applied for the transition layers being anywhere in the space). Note that Eq. 4 above corresponds to Eqs. S9-10 and S12 in Additional file 1. Through linear stability analysis, we found that this proposed state is indeed linearly stable in the case of *D* = 0 (see Additional file 1: Section 2).

When the system has a nonzero diffusion (*D* ≠ 0), we would like to determine if some perturbed form of the above steady state remains stable. This can be implemented by performing a perturbation expansion in *D*, i.e., $u_{i}∼u_{0i}+{\text{Du}}_{1i}+D^{2}u_{2i}+O(D^{3})$ and $v_{i}∼v_{0i}+{\text{Dv}}_{1i}+D^{2}v_{2i}+O(D^{3})$, where *u*_0*i*_ and *v*_0*i*_ are the steady state values presented in Eq. 4 (*u*_*i*_ and *v*_*i*_). To have a stable state, it is mandatory to have all *u*_*i*_ and *v*_*i*_ remain positive and bounded by the respective carrying capacities, which gives rise to a necessary condition for stability with *D*≠0 as (see Additional file 1: Section 2)

(5) $$\begin{array}{l} 1+c_{1}<\frac{\alpha }{\beta }<1+2c_{1} \end{array}$$

which corresponds to Eq. S20 in Additional file 1. The necessary condition also allows us to estimate the coexistence phase boundary in the limit as *D* → 0, as shown in Additional file 1: Figure S4

Although we only considered the perturbation results for two one-grid-point transition layers, our results can be generalized to larger transition layers. The simplicity of the one grid point case, however, provides an analytical bound between growth advantage and inhibition strength for the existence of stable localized patterns. As the diffusion constant of the model approaches to zero, we found that our analytical calculation from Eq. 5 indeed represents a good approximation of the phase boundaries obtained from numerical simulations (see in Additional file 1: Figure S4).

### Two dimensional patterns

Although we have primarily focused on the exploration of spatial population structures in one dimension thus far, coexistence and localized patterns can be present in a high dimensional space as well. To demonstrate our idea, we extended our model (Eq. 2) for the two dimensional case where both species diffusion and nearest neighbor interactions are over a square grid. Similar to the one dimensional case, an inhibition constant *c*_2_ = *c* / 5 was introduced to reflect the five locations for possible CDI (one on–site and four nearest neighbors).

**Figure 6:**
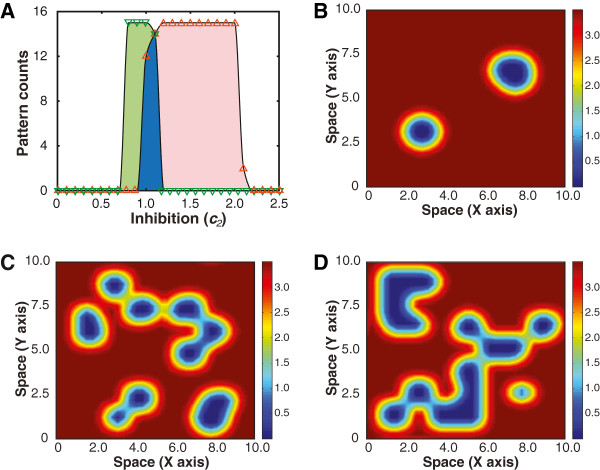
**Localized patterns in two dimensional space. (A)** Counts of stable localized patterns for two sets of initial conditions (see Additional file 1: Figure S9). The green and orange curves (triangles) correspond to the counts for the initial conditions when *u* is surrounded by *v* and *v* is surrounded by *u* respectively. The overlap region (blue) suggests a parameter regime where species coexistence is stable for both sets of initial conditions. **(B-D)** Localized patterns in two dimensional space with a growth advantage of 3.5, a diffusion constant (*D*) of 10^-3^, and an inhibition of 1.0, 1.05, and 1.1 respectively. Each pattern was chosen to represent the average space occupied by CDI- for 100 runs with random initial conditions. The average percentage of space occupied by CDI- for the three inhibition values is 95%, 84%, and 78% respectively.

To illustrate the plausibility of competition-induced spatial patterns, we examined possible outcomes of the population with the alteration of the strength of CDI inhibition while keeping the relative growth rate and diffusion constant fixed. Accordingly, two sets of representative initial conditions are employed: one set of initial conditions having a cluster of *u* that is centered in the space and surrounded by *v*, and the other set having a cluster of *v* centered in the space and surrounded by *u* (detailed in Methods and Additional file 1: Figure S9). Figure 6A shows the counts of stable coexistence patterns for both sets of the initial conditions with the green inverted triangles corresponding to the first set and the orange triangles corresponding to the second. It is clear that the counts of stable patterns are different for the two different initial condition sets even with the same parameter set, which is due to the asymmetry of the interactions between the CDI+ and CDI- species. By summing up the two counts, we found that the overlapping region of Figure 6A (blue region) corresponds to the parameter space where the population competition has the largest probability of forming coexisting patterns, corresponding to the least chance of species extinction, for the sets of initial conditions tested.

With the exploration of parameter space, we further illustrated spatially localized patterns in two dimensional space in Figure 6B-D where representative spatial distributions of species *u* are shown (see Additional file 1: Figure S5–7 for species *v*). The average amount of space occupied by species *u* decreases with the increase of the inhibition. As in the one dimensional case, the space occupied by *u* is larger than that of *v* in the coexistence region for the parameters explored. Our results demonstrate that the two dimensional model is indeed capable of producing coexistence for the two species with a rich set of possible patterned steady states.

## Conclusions and discussion

With a two-species population model, we have computationally investigated the dynamics and competition outcomes of a bacterial population with contact–dependent inhibition in different settings. We found that the tradeoff between the strength of inhibition via direct cell contact and relative growth advantage associated with metabolic burden are of central importance in determining the outcome of bacterial competition. In the well-mixed case, two competing species are mutually exclusive and their competition always leads to the extinction of one of the two species depending on the inhibition-growth tradeoff as well as initial conditions. In contrast, coexistence and localized patterns may also emerge from the competition of the population in the spatial case, in addition to exclusive extinctions. In addition, a statistical picture of a population with CDI-based competitions has been revealed, including the diversity, relative abundance, and pattern characteristics of all possible competition outcomes.

This study has hence expanded the spectrum of the current knowledge about contact dependent inhibition and provided a systematic view of CDI-based competition in bacterial populations by exploring possible outcomes in different settings. It also offers a quantitative calibration on the requirements for the emergence of various outcomes by examining the effects of inhibition, relative growth rate, and population diffusion. In addition, the population competition dynamics illustrated here increase our understanding of the complexity of bacterial social interactions. Furthermore, this work sheds light on new experimental design and tests by providing the predictions on spatial coexistence and localized patterns of the population structure as well as their initial condition dependence.

It is worthy to note that our mathematical model adopted a deterministic description of population competition. However, population dynamics and competition outcome may differ or, in some cases, deviate dramatically from those of a deterministic model when we take into account the stochastic nature of cellular growth [51]. For instance, diversity of emergent patterns and their characteristics may be altered significantly when the system is close to its phase boundary where multiple distinct spatial patterns occur. In addition, contact–dependent inhibition may have altered modes of action in different organisms as the expression of the CDI genes can be constitutive or stationary phase dependent [14,25,52]. For future study, it will therefore be valuable to consider the growth phase dependence of the activities of contact dependent inhibitions.

## Methods

### Numerical integration

To explore the competition outcomes of a two-species bacterial population, we numerically integrated our mathematical model consisting of a set of coupled ordinary differential equations (e.g., Eqs. 2 and 3). Here, integration was carried out using a Runge-Kutta adaptive step size method of order 4-5 [53,54]. The maximal error per step was set to 10^-6^ for all runs unless otherwise noted, and a maximal step size of 10^-1^ was imposed. To avoid negative values for any of the species concentrations during integration, we decreased the time step until all negative values were within the maximal error threshold from zero and subsequently set the negative values to zero before the next time step was taken. Our results for numerical integration were validated with random sample runs using a different maximal error and maximal step size as detailed in Additional file 1: Section 3.

Discretization of the reaction diffusion equations for our system was accomplished using a Taylor expansion approximation to the diffusion operator. Namely:

(6) $$\begin{array}{l} \frac{d^{2}u}{dx^{2}}\approx \frac{u(x+\delta )-2u(x)+u(x-\delta )}{\delta^{2}} \end{array}$$

which converts the coupled partial differential equations of our system to a set of coupled ordinary differential equations. The number of coupled ordinary differential equations was determined by the number of grid points employed for the system, which for the one dimensional case is 32 unless otherwise noted.

### Spatial patterns

To determine the possibility of population coexistence and aggregation in one dimensional space, we used initial conditions that involve two domains with each consisting solely of one of the two species at its individual carrying capacity as shown in Additional file 1: Figure S8. The domain size was varied between all possible sizes for each species with the entire space filled, which gives rise to 31 different initial conditions used with initial condition *j* given by:

(7) $$\begin{array}{lll} u_{i}=0 & v_{i}=\beta & i=1,\ldots ,j\\ u_{i}=\alpha & v_{i}=0 & i=j+1,\ldots ,32 \end{array}$$

where *j* varied from 1 to 31. This set of initial conditions was used to approximate the phase boundary for the coexistence of the two species. The boundary was further validated through the use of 10^4^ random initial conditions near the boundary for *α*/ *β*∈[3,4] and *D* = 10^-3^.

The statistics for patterns within the coexistence region were collected through the simulation of 10^4^ random initial conditions until convergence was reached. We considered the system to have converged if the maximal difference for all grids averaged below 10^-8^ times the largest carrying capacity in the system for 10^3^ time steps. The stripe widths were calculated by determining the dominant species at each grid point. Patterns were then classified according to the number of distinct stripes and widths present in the converged state.

We further extended our model to give illustrative examples of possible pattern formation in two dimensional space. To explore parameter space, we utilized a set of initial conditions in which each species formed a spatial aggregate at the center of space surrounded by a sea of its competitor. For example, a square of 4 grid points of species *u* at its carrying capacity was surrounded by species *v* at its carrying capacity. The interior species size ranged from 4 grid points to 256 grid points with the total system size set at 1024 grid points (see Additional file 1: Figure S9).

### Parameter space

For the well–mixed case, the phase diagram (Figure 1A) was determined using linear stability analysis. The Lyapunov function [36] in the either extinction region was constructed using *α* = 2, *β* = 1, and *c* = 2 (Figure 1B). The representative time course for the parameter regions employed *α* = 2, *β* = 1, and *c* = 0.5 (Figure 1C), *α* = 0.5, *β* = 1, and *c* = 2 (Figure 1D), and *α* = 2, *β* = 1, and *c* = 2 (Figure 1E).

In Figure 2, an identical set of initial conditions was used to produce the spatial patterns. The growth advantage (*α* / *β*) and inhibition (*c*_1_) were held fixed at 3.5 and 2.1 respectively, while *D* was varied between 10^-3^, 10^-2^, and 10^-1^ for Figure 2A–C respectively.

To systematically explore the tradeoff between relative growth advantage and inhibition in the small diffusion limit, we searched over parameter space in the one dimensional model with *α*/*β* ∈ [1, 5], *c*_1_ ∈ [0, 4], and *D* ∈ {10^-4^, 10^-3^, 10^-2^} to generate the phase diagram in Figure 3A. The growth advantage and inhibition strength were sampled every 10^-1^ here. For Figure 3B–G, a growth advantage (*α*/ *β*) of 3.5 was used with a varying inhibition strength (*c*_1_), given by 1.7, 1.8, 1.9, 2.0, 2.2, 2.3 respectively, to find the most likely steady state out of 10^4^ random initial conditions.

In Figure 4, we used a growth advantage (*α*/ *β*) of 3.5, an inhibition constant (*c*_1_) of 2.2, and a diffusion constant of 10^-3^. The percentages were taken from 10^4^ random initial conditions in which the steady states were classified according to single stripe, double stripe, multi–stripe, or extinction.

As a summary of the types of patterns observed across the coexistence region in the parameter space, Figure 5 shows the relative abundance of patterns when varying growth advantage and inhibition. In Figure 5A, the growth advantage and diffusion constant are fixed at 3.5 and 0.001 respectively while the inhibition is varied between 1.4 and 2.3 with 0.1 increments. In Figure 5B, the inhibition and diffusion constant are fixed at 2.0 and 0.001 respectively while the growth advantage is varied between 3.2 and 4.6 with 0.1 increments.

For the two dimensional model, *α*/ *β* and *D* were kept fixed at 3.5 and 10^-3^ respectively while *c*_2_ was sampled every 10^-1^ within [0,3]. Figure 6A was generated using a set of initial conditions with each species surrounded by a sea of its competitor at carrying capacity (see Additional file 1: Figure S9). Figure 6B–D are possible steady state patterns using an inhibition constant (*c*_2_) of 1.0, 1.05, and 1.1 respectively.

### Analytical results

Analytical results for the phase boundaries were determined using asymptotic expansions and linear stability analysis (see Additional file 1: Section 2 for details). For an introduction to asymptotic expansions and linear stability, see [55]. The existence of a stable coexistence state for slow diffusion in discrete systems was proposed in [29] for a two grid point system through the use of a perturbation theorem that remains valid for any number of grid points.

## Competing interests

The authors declare that they have no competing interests.

## Authors’ contributions

The project was conceived by TL, AB, and VC. AB wrote the Matlab code for numerical integration, carried out the simulations, and analyzed the resulting data. AB performed the analytical calculations to establish stability of steady states and perturbative corrections for the model. TL and AB processed the data to produce all figures. TL, AB, and VC prepared the manuscript. TL coordinated the project. All authors read and approved the final manuscript.

## Supplementary Material

Additional file 1 Supplementary Information. Click here for file
