# Supplementary material for: Extinction, coexistence, and localized patterns of a bacterial population with contact-dependent inhibition
Source: BMC Syst Biol. 2014 Feb 27;8:23. doi: 10.1186/1752-0509-8-23 (PMC3942258; doi:10.1186/1752-0509-8-23)
Supplement: Additional file 1 — Supplementary Information. [file 1752-0509-8-23-S1.pdf]

Supplementary Information for Extinction, Coexistence, and Localized Patterns of a Bacterial Population with Contact-Dependent Inhibition

Andrew Blanchard, Venhar Celik, and Ting Lu
Department of Bioengineering, Department of Physics, and Institute for Genomic Biology
University of Illinois at Urbana-Champaign, Urbana, IL 61801, U.S.A.

1 Mathematical Competition Model

We propose the following model to capture the interactions of two competing bacterial species with one employing CDI.

$$\begin{aligned}\frac{du}{dt} &= u\left(a - b(u+v) - c\int f(|\chi - x|)v(\chi)d\chi\right) + D_u\nabla^2 u\\ \frac{dv}{dt} &= v(d - b(u+v)) + D_v\nabla^2 v\end{aligned} \tag{S1}$$

Notice that for simplicity we have assumed that u and v compete for nutrients in the same way (coefficient b). We now remove the dimensions from the system by introducing the following variables:

$$\begin{aligned}u &= u_c\overline{u}\\ v &= v_c\overline{v}\\ t &= t_c\overline{t}\end{aligned} \tag{S2}$$

Plugging these new variables into our equations (and dropping the bars) gives:

$$\begin{aligned}\frac{du}{dt} &= t_c u\left(a - b(u_c u + v_c v) - c v_c\int f(|\chi - x|)v(\chi)d\chi\right) + t_c D_u\nabla^2 u\\ \frac{dv}{dt} &= t_c v(d - b(u_c u + v_c v)) + t_c D_v\nabla^2 v\end{aligned} \tag{S3}$$

We can now choose the dimensions of our system to eliminate some of the quantities. We choose the following way to remove the dimensions from the system:

$$\begin{aligned}t_c b u_c &= 1\\ t_c b v_c &= 1\\ t_c d &= 1\end{aligned} \tag{S4}$$

With these choices, our equations become:

$$\begin{aligned}\frac{du}{dt} &= u\left(\alpha - u - v - \overline{c}\int f(|\chi - x|)v(\chi)d\chi\right) + \overline{D_u}\nabla^2 u\\ \frac{dv}{dt} &= v(1 - u - v) + \overline{D_v}\nabla^2 v\end{aligned} \tag{S5}$$

where we have:

$$\alpha = \frac{a}{d}$$
$$\overline{c} = \frac{c}{b}$$
$$\overline{D_u} = \frac{D_u}{d}$$
$$\overline{D_v} = \frac{D_v}{d} \quad \text{(S6)}$$

Notice that we have left our spatial coordinate x with units. We could non-dimensionalize the spatial dimension as well (e.g. by taking $x = L\overline{x}$), but this would just amount to a rescaling of our diffusion and inhibition coefficients. Getting rid of the bars, we get our model equation from the paper:

$$\begin{aligned} \frac{du}{dt} &= u\left(\alpha - u - v - c\int f(|\chi - x|)v(\chi)d\chi\right) + D_u\nabla^2 u \\ \frac{dv}{dt} &= v(\beta - u - v) + D_v\nabla^2 v \end{aligned} \quad \text{(S7)}$$

where we can set $\beta = 1$ without loss of generality. See [1] for an introduction to bacterial competition models and non–dimensionalization.

2 Stability of the One Grid Transition Layer State

For the discretized version of our model, namely:

$$\begin{aligned} \frac{du_i}{dt} &= u_i(\alpha - u_i - v_i - c_1(v_{i+1} + v_i + v_{i-1})) + \frac{D}{\delta^2}(u_{i+1} - 2u_i + u_{i-1}) \\ \frac{dv_i}{dt} &= v_i(\beta - u_i - v_i) + \frac{D}{\delta^2}(v_{i+1} - 2v_i + v_{i-1}) \\ i &= 1, 2, ..., N \end{aligned} \quad \text{(S8)}$$

where $c_1 = c/3$, we investigate the stability of a certain steady state for $D = 0$ in which spatial aggregation occurs. The simplest of such states involves one stripe of each species with two one-grid transition layers. Within the stripes, the species have the form:

$$u_i = \alpha \quad v_i = 0$$
$$\text{or}$$
$$u_i = 0 \quad v_i = \beta \quad \text{(S9)}$$

In the one-grid transition layers, the species have the form:

$$u_i = \alpha - \beta c_1 \quad v_i = 0 \quad \text{(S10)}$$

If we consider the case where the number of grid points N is larger than 6 and the system is divided evenly amongst the high u state and the high v state with two transition layers, we can write down the eigenvalues of our system and their degeneracy. The set of all eigenvalues is: $\lambda = \{-\alpha, \beta - \alpha, -\beta, \alpha - \beta(1 + 3c_1), -\alpha + \beta c_1, -\alpha + \beta(1 + c_1), \alpha - \beta(1 + 2c_1)\}$. If we define $n = (2N - 12)/4$, then the degeneracies are: $\{n + 2, n + 2, n + 2, n, 2, 2, 2\}$. Thus, in the parameter

region where we find patterns, this state is linearly stable, and the perturbation theorem [2] applies and tells us that some perturbed state remains stable for small diffusion.

All but two of the eigenvalues can be found easily for the one grid transition layer by inspection. The sum of the remaining two can also be calculated using the trace of the matrix. Numerical validation was then used to find the remaining two eigenvalues, which are in fact $-\beta$.

We now wish to find out what happens to this solution when $D \neq 0$. For convenience, we will absorb the δ^2 into D in the following. To do this, we propose a perturbation expansion (see [3] for an introduction) about our single transition layer steady state solution for small D.

$$\begin{aligned} u_i &\sim u_{0i} + Du_{1i} + D^2 u_{2i} + ... \\ v_i &\sim v_{0i} + Dv_{1i} + D^2 v_{2i} + ... \end{aligned} \tag{S11}$$

Where we use the following form for u_{0i} and v_{0i}:

$$\begin{aligned} &u_{0i} = \alpha && v_{0i} = 0 && i = 1, ..., \frac{N}{2} - 1 \\ &u_{0i} = \alpha - \beta c_1 && v_{0i} = 0 && i = \frac{N}{2} \\ &u_{0i} = 0 && v_{0i} = \beta && i = \frac{N}{2} + 1, ...N - 1 \\ &u_{0i} = \alpha - \beta c_1 && v_{0i} = 0 && i = N \end{aligned} \tag{S12}$$

which corresponds to transition layers at $i = N/2$ and $i = N$. Our approach should be generic for two transition layers at any grid points provided that there is sufficient spacing between the layers. We now write the equation to order $\mathcal{O}(D)$.

$$\begin{aligned} u_{0i}(-u_{1i} - v_{1i}(1 + c_1) - c_1(v_{1i+1} + v_{1i-1})) + u_{1i}(\alpha - u_{0i} - v_{0i}(1 + c_1) - c_1(v_{0i+1} + v_{0i-1})) \\ + (u_{0i+1} - 2u_{0i} + u_{0i-1}) = 0 \\ v_{0i}(-u_{1i} - v_{1i}) + v_{1i}(\beta - u_{0i} - v_{0i}) + (v_{0i+1} - 2v_{0i} + v_{0i-1}) = 0 \end{aligned} \tag{S13}$$

Away from the boundaries, these equations simplify drastically due to the homogeneous nature of the solution. Specifically, for $i = 1, ..., N/2 - 1$ we get:

$$v_{1i}(\beta - \alpha) = 0 \tag{S14}$$

which tells us that $v_{1i} = 0$ for this range. Using the result we find that for $i = 2, ..., N/2 - 2$

$$\alpha(-u_{1i}) = 0 \tag{S15}$$

Thus, u_{1i} is zero over this range. Furthermore, over the range $i = N/2 + 2, ..., N - 2$ we get:

$$u_{1i}(\alpha - \beta(1 + 3c_1)) = 0 \tag{S16}$$

Thus, u_{1i} is zero over this range. This implies that v_{1i} is zero over the same range. Thus, we have the following terms left to find:

$$\begin{aligned} &u_{1i} && i = 1, \frac{N}{2} - 1, \frac{N}{2}, \frac{N}{2} + 1, N - 1, N \\ &v_{1i} && i = \frac{N}{2}, \frac{N}{2} + 1, N - 1, N \end{aligned} \tag{S17}$$

We begin with some simple equations:

$$\begin{aligned} v_{1N}(\beta - \alpha + \beta c_1) + \beta = 0 \\ v_{1\frac{N}{2}}(\beta - \alpha + \beta c_1) + \beta = 0 \\ u_{1N-1}(\alpha - \beta(1 + c_1) - \beta c_1) + (\alpha - \beta c_1) = 0 \\ u_{1\frac{N}{2}+1}(\alpha - \beta(1 + c_1) - \beta c_1) + (\alpha - \beta c_1) = 0 \end{aligned} \tag{S18}$$

Let's consider the solutions to two of these equations:

$$\begin{aligned} u_{1N-1} &= \frac{-(\alpha - \beta c_1)}{\alpha - \beta(1 + 2c_1)} \\ v_{1N} &= \frac{\beta}{\alpha - \beta(1 + c_1)} \end{aligned} \tag{S19}$$

The requirement that u and v be positive to first order in D/δ^2 tells us that:

$$1 + c_1 < \frac{\alpha}{\beta} < 1 + 2c_1 \tag{S20}$$

Furthermore, as α/β approaches the boundaries of the above inequalities, our perturbation expansion breaks down. We present all of the terms for one boundary condition below. The other boundary is the same by symmetry.

$$\begin{aligned} u_{11} &= -\left(\frac{\beta c_1}{\alpha} + \frac{\beta c_1}{\alpha - \beta(1 + c_1)}\right) \\ v_{11} &= 0 \\ u_{1N} &= -v_{1N}(1 + c_1) - c_1 v_{1N-1} + \frac{-\alpha + 2\beta}{\alpha - \beta c_1} \\ v_{1N} &= \frac{\beta}{\alpha - \beta(1 + c_1)} \\ u_{1N-1} &= \frac{-(\alpha - \beta c_1)}{\alpha - \beta(1 + 2c_1)} \\ v_{1N-1} &= -u_{1N-1} - 1 \end{aligned} \tag{S21}$$

3 Validation of Numerical Methods

To validate the numerical procedure used to integrate the differential equations of our model, we have validated our Runge–Kutta adaptive step size methods with Matlab's ode45 solver and tested our steady state results using a different error tolerance and maximum step width.

As an initial validation, our ode solver was tested with ode45 in three cases: exponential decay, homogeneous two species competition, and a three species system with diffusion and only onsite interactions. In all cases, the difference between the two solvers was on the order of 10^{-5} or smaller for an error tolerance of 10^{-6}.

To test the effects of changing the error tolerance and the maximum step size on our results, we tested the steady state values for our solver for 105 different patterned states from our investigation of phase space. Using an error tolerance of 10^{-12} resulted in negligible changes to the steady states, with differences from the 10^{-6} tolerance smaller than 10^{-15}. Using a maximum step size of 10^{-2} instead of 10^{-1} with an error tolerance of 10^{-6} also resulted in negligible changes to the steady states with differences smaller than 10^{-13}

4 Supplementary Figures and Tables

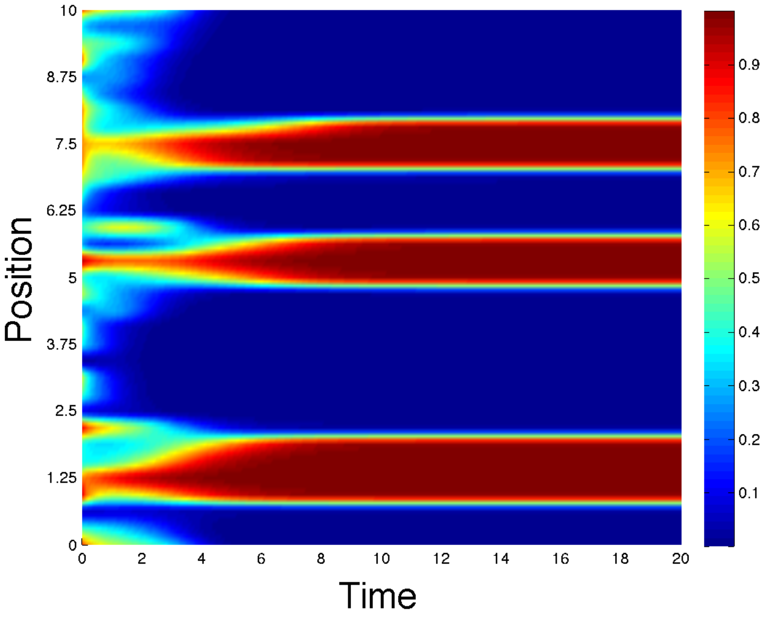

Figure S1: Corresponding figure for species v to main text figure 2A.

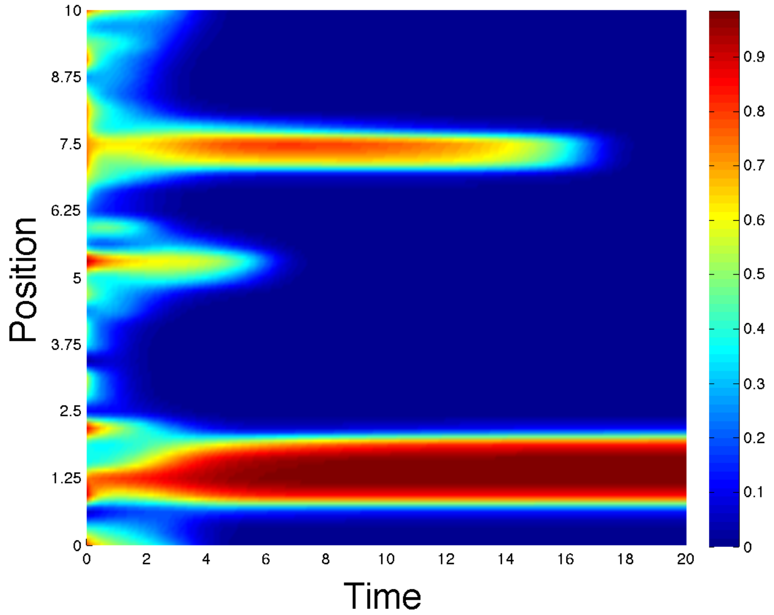

Figure S2: Corresponding figure for species v to main text figure 2B.

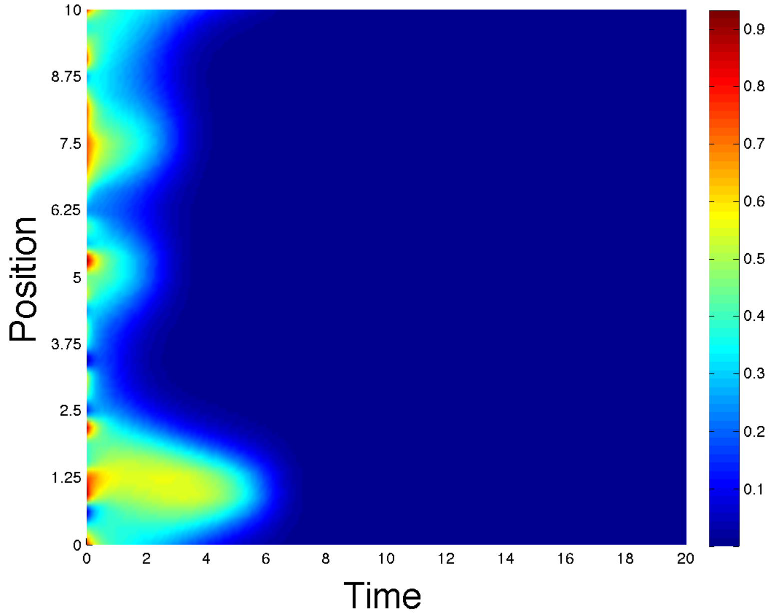

Figure S3: Corresponding figure for species v to main text figure 2C.

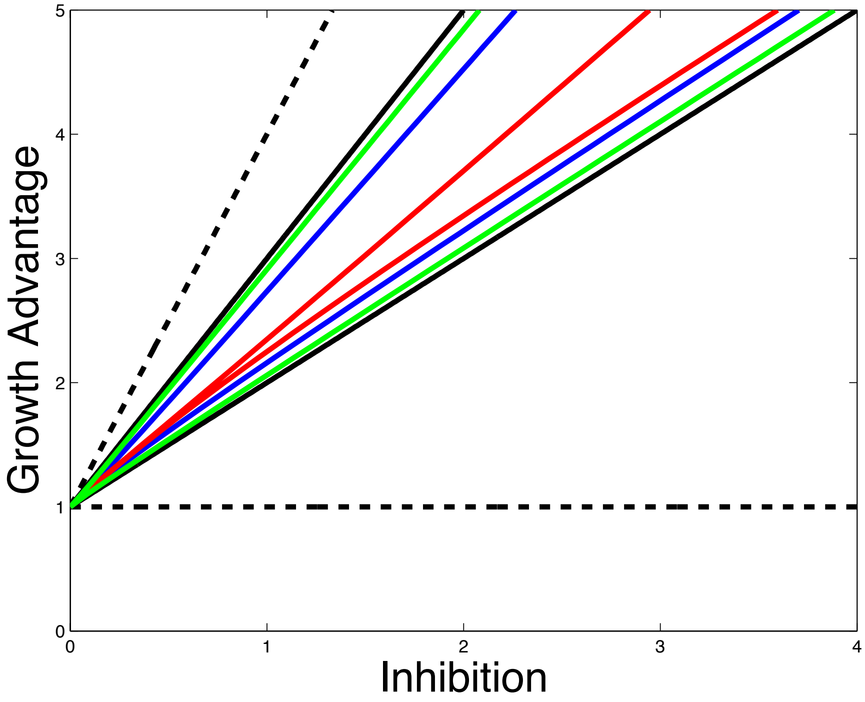

Figure S4: Corresponding to figure 3A in the main text, the phase diagram for spatial coexistence with different values of diffusion. Growth advantage is α/β and inhibition is c_1. The red ($D = 0.01$), blue ($D = 0.001$), and green ($D = 0.0001$) lines were shown in the main text along with the dotted lines for stability of both extinction states in the well–mixed case. The black line is the calculated stability region for the one grid transition layer state discussed in the Perturbation Expansion for Patterned States section of the main text. Notice that the phase boundaries approach the black solid line as $D \to 0$.

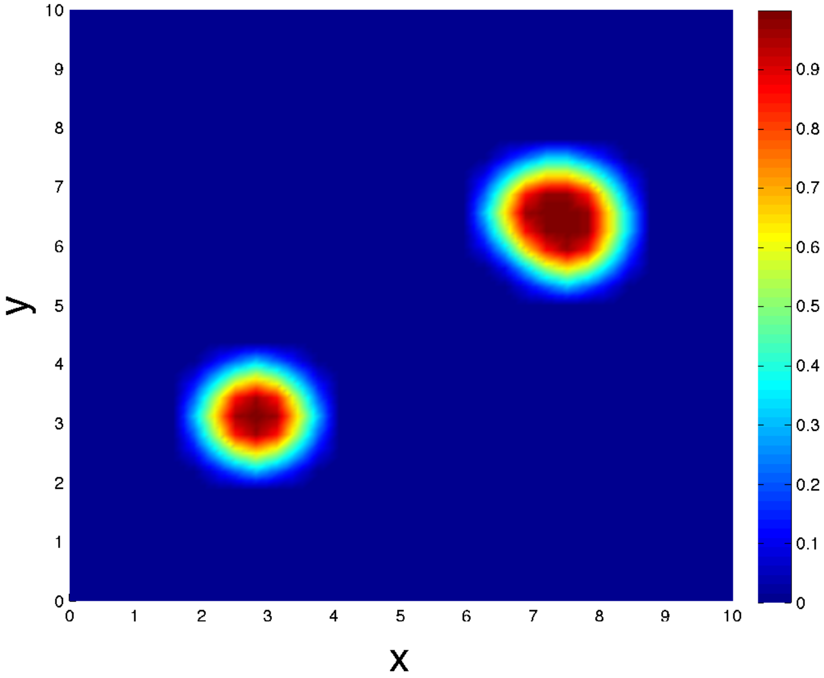

Figure S5: Corresponding figure for species v to main text figure 6B.

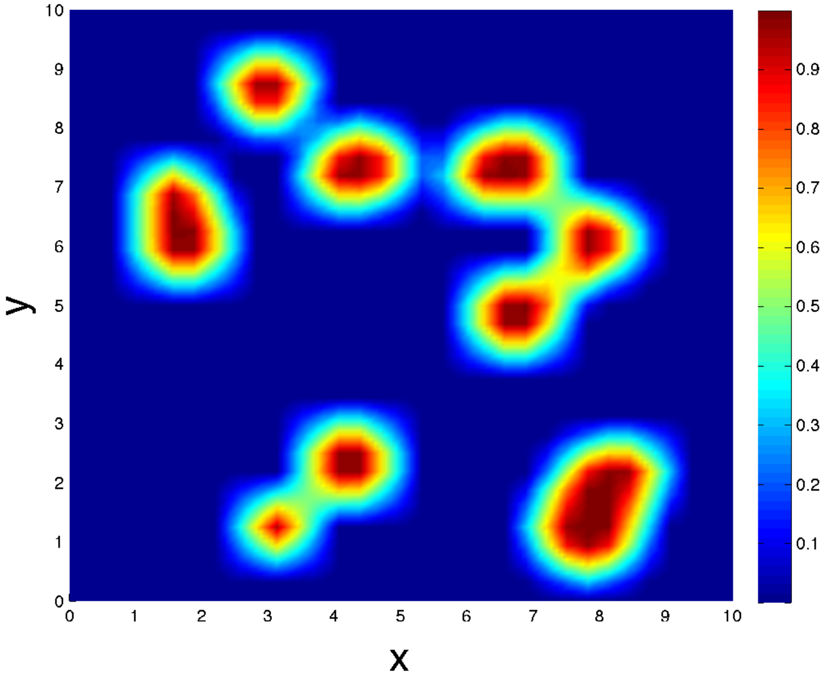

Figure S6: Corresponding figure for species v to main text figure 6C.

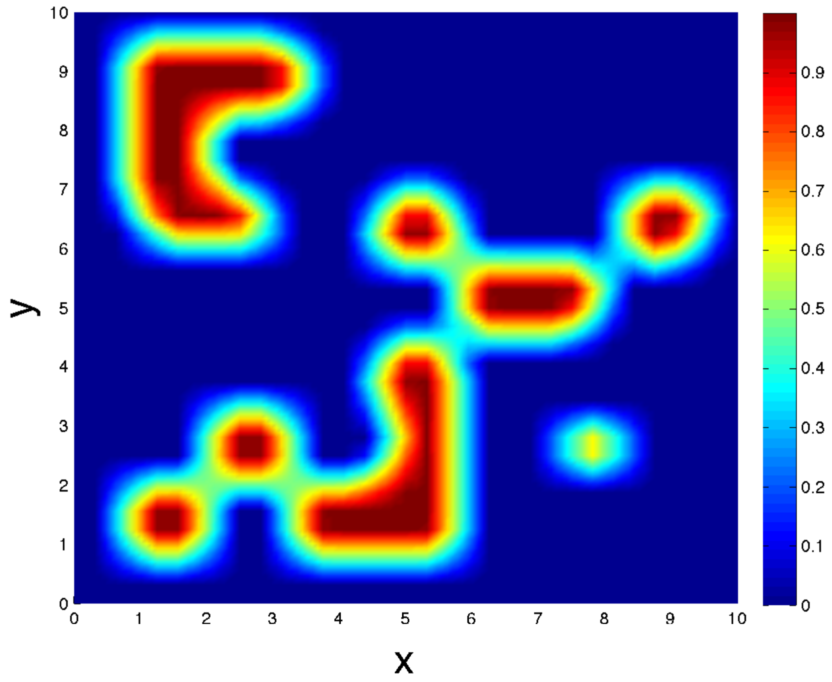

Figure S7: Corresponding figure for species v to main text figure 6D.

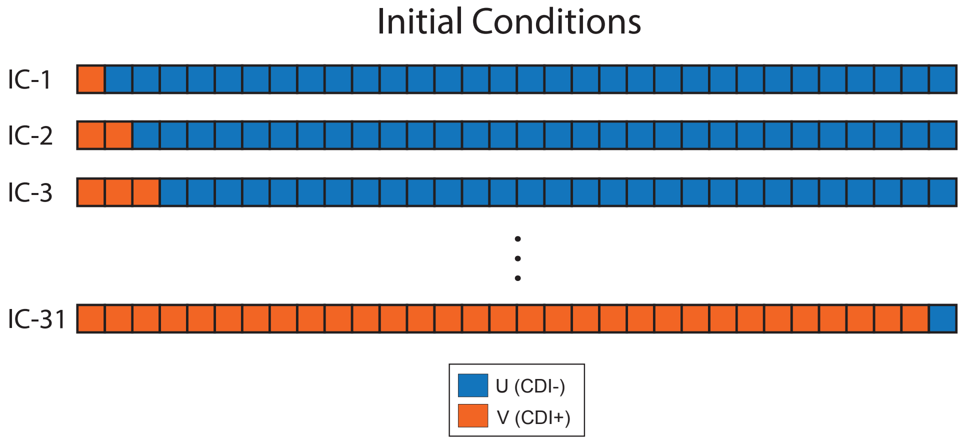

Figure S8: Initial conditions used to determine the phase diagram for coexistence (fig. 3A). Each grid consists of one species at its respective carrying capacity.

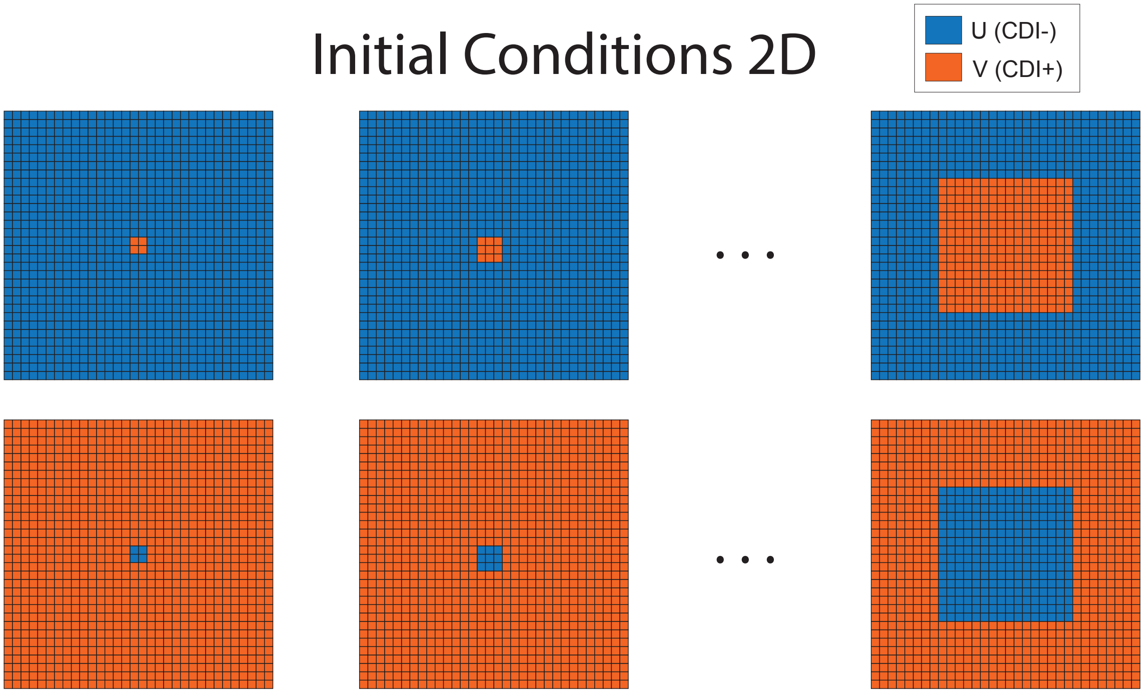

Figure S9: Initial conditions used to determine the parameters for possible coexistence in two dimensions (fig. 6A). Each grid consists of one species at its respective carrying capacity.

c_1	Single Stripe %	Double Stripe %	Multi–Stripe %	Extinct U %	Extinct V %
1.4000	0.0000	0.0000	0.0000	0.0000	100.0000
1.5000	4.0100	0.0300	0.0000	0.0000	95.9600
1.6000	25.0500	3.1400	0.1500	0.0000	71.6600
1.7000	38.4000	9.6500	1.2500	0.0000	50.7000
1.8000	42.8500	20.9200	4.3200	0.0000	31.9100
1.9000	40.3400	31.9200	10.4900	0.0000	17.2500
2.0000	32.7800	38.9900	19.8100	0.0000	8.4200
2.1000	24.1800	42.8300	29.0500	0.0000	3.9400
2.2000	23.7100	51.4700	23.3400	0.0900	1.3900
2.3000	0.0000	0.0000	0.0000	99.3400	0.6600

Table 1: Summary of statistics for different inhibition values (c_1) using a growth advantage (α/β) of 3.5 and a diffusion constant (D) of 0.001.This table was used to generate figure 5A.

α/β	Single Stripe %	Double Stripe %	Multi–Stripe %	Extinct U %	Extinct V %
3.2000	0.0000	0.0000	0.0000	99.4700	0.5300
3.3000	23.6600	51.7100	22.7600	0.1700	1.7000
3.4000	24.7900	43.2200	28.1900	0.0000	3.8000
3.5000	33.0700	39.0500	19.8300	0.0000	8.0500
3.6000	39.4700	32.7400	11.7400	0.0000	16.0500
3.7000	44.1000	23.9800	6.2900	0.0000	25.6300
3.8000	41.6800	16.4800	2.9800	0.0000	38.8600
3.9000	37.8900	9.3800	1.1900	0.0000	51.5400
4.0000	30.3600	4.6800	0.4000	0.0000	64.5600
4.1000	21.1600	2.0800	0.1000	0.0000	76.6600
4.2000	13.8500	0.8500	0.0800	0.0000	85.2200
4.3000	7.6800	0.2100	0.0000	0.0000	92.1100
4.4000	1.1700	0.0000	0.0000	0.0000	98.8300
4.5000	0.5300	0.0000	0.0000	0.0000	99.4700
4.6000	0.0000	0.0000	0.0000	0.0000	100.0000

Table 2: Summary of statistics for different growth advantage values (α/β) using an inhibition (c_1) of 2.0 and a diffusion constant (D) of 0.001. This table was used to generate figure 5B.

c_1	Single Stripe Width	Double Stripe Width	Multi–Stripe Width
1.5000	1.0793±0.2727	1.0417±0.1473	0.0000
1.6000	0.8282±0.3142	0.8056±0.3052	0.7708±0.2764
1.7000	0.9027±0.4039	0.8847±0.3750	0.8251±0.3011
1.8000	1.0117±0.5083	0.9700±0.4509	0.9135±0.4012
1.9000	1.1579±0.6373	1.0832±0.5682	0.9980±0.4637
2.0000	1.4131±0.8970	1.2581±0.7115	1.1059±0.5645
2.1000	1.8091±1.2791	1.5165±0.9220	1.2350±0.6551
2.2000	3.2307±2.1881	1.9789±1.2558	1.3787±0.7482

Table 3: Corresponding table for species v, see Table 1 in the main text

α/β	Single Stripe Width	Double Stripe Width	Multi–Stripe Width
3.3000	3.3140±2.2685	1.9957±1.2667	1.3985±0.7617
3.4000	1.7604±1.2199	1.5108±0.9345	1.2359±0.6529
3.5000	1.3780±0.8392	1.2885±0.7402	1.1180±0.5645
3.6000	1.1701±0.6749	1.1191±0.5964	1.0318±0.4983
3.7000	1.0585±0.5602	1.0053±0.4879	0.9399±0.4229
3.8000	0.9456±0.4490	0.9315±0.4266	0.9084±0.3862
3.9000	0.8897±0.3828	0.8725±0.3645	0.8200±0.2919
4.0000	0.8381±0.3433	0.8083±0.2990	0.7929±0.2814
4.1000	0.8047±0.2962	0.8226±0.3324	0.8229±0.2851
4.2000	0.7881±0.2598	0.7279±0.2111	0.7682±0.2850
4.3000	0.7690±0.2421	0.8259±0.2712	0.0000
4.4000	1.0443±0.2050	0.0000	0.0000
4.5000	1.0377±0.2083	0.0000	0.0000

Table 4: Corresponding table for species v, see Table 2 in the main text

References

[1] Murray, J.D.: Mathematical Biology. Springer, New York (2002)

[2] Levin, S.: Dispersion and population interactions. American Naturalist **108**(960), 207–228 (1974)

[3] Holmes, M.H.: Introduction to Perturbation Methods. Springer, New York (2013)
